# Antipsoriatic Potential of Quebecol and Its Derivatives

**DOI:** 10.3390/pharmaceutics14061129

**Published:** 2022-05-26

**Authors:** Corinne Bouchard, Alexe Grenier, Sébastien Cardinal, Sarah Bélanger, Normand Voyer, Roxane Pouliot

**Affiliations:** 1 Centre de Recherche en Organogénèse Expérimentale de L’Université Laval/LOEX, Axe Médecine Régénératrice, Centre de Recherche du CHU de Québec-Université Laval, Quebec, QC GIJ 1Z4, Canada; corinne.bouchard.2@ulaval.ca (C.B.); alexe.grenier.1@ulaval.ca (A.G.); sarah.belanger.5@ulaval.ca (S.B.); 2 Faculté de Pharmacie, Université Laval, Quebec, QC G1V 0A6, Canada; 3 Département de Biologie, Chimie et Géographie, Université du Québec à Rimouski, Rimouski, QC G5L 3A1, Canada; sebastien_cardinal@uqar.ca; 4 Département de Chimie and PROTEO, Faculté des Sciences et Génie, Université Laval, Quebec, QC G1V 0A6, Canada; normand.voyer@chm.ulaval.ca

**Keywords:** skin substitutes, in vitro culture, psoriasis, antipsoriatic treatment, quebecol, polyphenol, maple syrup

## Abstract

Psoriasis is a chronic inflammatory skin disease mainly characterized by the hyperproliferation and abnormal differentiation of the epidermal keratinocytes. An interesting phenolic compound, namely quebecol (2,3,3-tri-(3-methoxy-4-hydroxyphenyl)-1-propanol) (compound **1**, CPD1), was isolated from maple syrup in 2011 and was recently synthesized. Quebecol and its derivatives ethyl 2,3,3-tris(3-hydroxy-4-methoxyphenyl)propenoate (compound **2**, CPD2) and bis(4-hydroxy-3-methoxyphenyl)methane (compound **3**, CPD3) have shown antiproliferative and anti-inflammatory potential, making them promising candidates for the treatment of psoriasis. This study aimed to evaluate the antipsoriatic potential of quebecol and its derivatives on psoriatic skin substitutes produced according to the self-assembly method. A sulforhodamine B (SRB) assay determining the concentration that inhibits 20% of cell growth (IC_20_) was performed for CPD1, CPD2 and CPD3, and their IC_20_ values were 400, 150 and 350 μM, respectively. At these concentrations, cell viability was 97%, 94% and 97%, respectively. The comparative control methotrexate (MTX) had a cell viability of 85% at a concentration of 734 μM. Histological analyses of psoriatic skin substitutes treated with CPD1, CPD2 and CPD3 exhibited significantly reduced epidermal thickness compared with untreated psoriatic substitutes, which agreed with a decrease in keratinocyte proliferation as shown by Ki67 immunofluorescence staining. The immunofluorescence staining of differentiation markers (keratin 14, involucrin and loricrin) showed improved epidermal differentiation. Taken together, these results highlight the promising potential of quebecol and its derivatives for the treatment of psoriasis.

## 1. Introduction

Psoriasis is an erythemato-squamous skin disease affecting about 2–3% of the world’s population [1]. This chronic inflammatory dermatosis is characterized by histopathological features including acanthosis, hyperkeratosis, parakeratosis, hypogranulosis or agranulosis, papillomatosis, the infiltration of immune cells and angiogenesis [2,3,4]. The etiology of psoriasis still remains unknown, which is why it can be difficult to find an appropriate treatment for patients. Moreover, for the exact same reason, there is still no curative treatment at the moment. Psoriasis is a multifactorial pathology that could benefit from new treatments to help patients control their symptoms without any drawbacks. A considerable portion of patients are not satisfied with their current treatment, mainly due to the undesirable side effects of treatments on the market or a lack of efficacy [5]. In a Scandinavian study, 30.5% of the patients were dissatisfied with systemic treatments, especially methotrexate, a common psoriasis treatment [6]. In the same study, 22.9% of respondents were not satisfied with biologic treatments. These findings demonstrate the importance of finding new antipsoriatic agents that could decrease symptoms and increase the patients’ quality of life.

**Figure 1:**
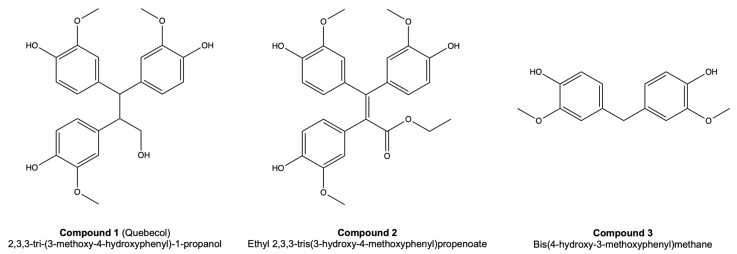
Structure of quebecol (2,3,3-tri-(3-methoxy-4-hydroxy-phenyl)-1-propanol) (compound **1**, CPD1), ethyl 2,3,3-tris(3-hydroxy-4-methoxyphenyl)propenoate (compound **2**, CPD2) and bis(4-hydroxy-3-methoxyphenyl)methane (compound **3**, CPD3).

Maple syrup, a major product of the food industry in Canada, and more specifically in Quebec, has been the subject of several studies in the past years. Maple syrup, or phenolic compounds contained in maple syrup, were found to have in vitro antiproliferative, antioxidant and anti-inflammatory activities [7,8,9,10]. These findings led to the investigation and identification of the compounds contained in maple syrup. While studying the chemical composition of Canadian maple syrup, Seeram and his team discovered a novel phenolic compound: 2,3,3-tri-(3-methoxy-4-hydroxyphenyl)-1-propanol, also known as quebecol [11]. In order to compensate for its low natural abundance, Voyer’s research group (Université Laval) succeeded in achieving the complete synthesis of quebecol, in its racemic version [12]. Quebecol and its derivatives have also been the subject of several investigations, such as studies of their biological activity. Those studies put forward the antiproliferative and anti-inflammatory potential (on various cancer cell lines, and on macrophage and monocyte cell lines, respectively) of quebecol and derivatives, leading to an interest in these compounds for the treatment of psoriasis [13,14]. Moreover, prior to this study, our team screened quebecol and several of its derivatives in order to select the most promising antipsoriatic agents. The aim of this study was to evaluate the antipsoriatic potential of quebecol (compound **1**, CPD1), its synthetic analog ethyl 2,3,3-tris(3-hydroxy-4-methoxyphenyl)propenoate (compound **2**, CPD2) and its substructure bis(4-hydroxy-3-methoxyphenyl)methane (compound **3**, CPD3) (Figure 1) using an in vitro psoriatic skin model. Methotrexate (MTX) was used as a reference compound since it is commonly used to treat moderate to severe psoriasis. In that regard, the efficacy of the three compounds on the physiopathology of psoriasis, and more specifically on acanthosis, was assessed for the first time on a model mimicking psoriasis. Histological analyses and the expression of proliferation and differentiation markers as determined by immunofluorescence staining were used to compare psoriatic skin substitutes treated with CPD1, CPD2 or CPD3 with the non-treated psoriatic skin substitutes.

## 2. Materials and Methods

### 2.1. Synthesis of CPD1, CPD2 and CPD3 

The synthesis of the three compounds has previously been described in detail elsewhere [12,15].

### 2.2. Biological Testing

On the day of the treatments, stock solutions were prepared by diluting the compounds in DMSO due to the insolubility of the compounds in cell culture media. Then, the stock solutions were added to cell culture media. The final concentration of DMSO in the culture medium was maintained at a maximum of 0.4% (*v*/*v*) to avoid solvent cytotoxicity. Methotrexate (MTX; Injectable USP Methotrexate, 25 mg/mL, Hospira, Montréal, QC, Canada), commonly recommended to treat psoriasis, was used as a reference compound. To administer a typical starting dose of MTX for treating severe psoriasis, with a current recommended dose of 7.5 to 25 mg per week, a concentration of 734 μM was used, representing three doses of 6.67 mg or 20 mg per week [16,17]. This concentration previously demonstrated a similar response with the skin substitutes to what was observed with the skin in vivo [18]. MTX was added directly to the fresh culture medium.

### 2.3. Biopsies

Healthy skin substitutes (HSs) were reconstructed with cells from biopsies obtained during breast reduction surgeries. Donors were Caucasian females aged between 18 and 49 years old (♀18, ♀46 and ♀49). As for the psoriatic skin substitutes (PSs), they were reconstructed with cells from lesional psoriatic biopsies of donors in various stages of plaque-type psoriasis. Psoriatic donors were Caucasian males and females aged between 46 and 64 years old (♂46, ♂49 and ♀64).

### 2.4. Cell Extraction

Cells were extracted from the biopsies (healthy or psoriatic) using the isolation method described previously [19,20]. Fibroblasts were extracted using the isolation method with thermolysin and collagenase, while keratinocytes were extracted using the isolation method with thermolysin and trypsin. The cells were then cultured to the desired passage and frozen in liquid nitrogen until needed for the experiments. Fibroblasts were used at passage 4 and keratinocytes at passage 1.

### 2.5. Cell Culture

Fibroblasts (passage 4) were seeded at 4 × 10^3^ cells/cm^2^ and cultured in Dulbecco’s Modified Eagle’s Medium (DMEM) supplemented with 10% fetal calf serum (FCS, Invitrogen, Burlington, ON, Canada), 100 IU/mL penicillin G (Sigma, Oakville, ON, Canada) and 25 μg/mL gentamicin (Schering, Pointe-Claire, QC, Canada). Keratinocytes (passage 1) were seeded at 4 × 10^3^ cells/cm^2^ on a feeder layer of irradiated 3T3 murine fibroblasts and cultured in a combination of DMEM with Ham’s F12 (3:1) (DMEMH), supplemented with 5% Fetal Clone II serum (Hyclone, Scarborough, ON, Canada), 5 μg/mL insulin (Sigma, Oakville, ON, Canada), 0.4 μg/mL hydrocortisone (Galenova, Saint-Hyacinthe, QC, Canada), 10^−10^ M cholera toxin (MP Biomedicals, Montreal, QC, Canada), 10 ng/mL human epidermal growth factor (EGF; Austral Biological, San Ramon, CA, USA), 100 IU/mL penicillin G (Sigma) and 25 μg/mL gentamicin (Schering). All cell cultures were incubated at 37 °C in an 8% carbon dioxide (CO_2_) atmosphere, and the cell culture medium was changed three times per week.

### 2.6. Sulforhodamine B Assay

The cell growth inhibition was determined using the sulforhodamine B (SRB) assay with some minor modifications [21]. Briefly, healthy and psoriatic keratinocytes at passage 2 were seeded in a 96-well plate at 1 × 10^4^ cells/well on a feeder layer of irradiated 3T3 murine fibroblasts. On day 2, keratinocytes were treated with increasing concentrations of the compounds and incubated for 48 h (37 °C, 8% CO_2_). On day 4, cells were fixed with a 50% solution of trichloroacetic acid (TCA) at 4 °C for 60 min to stop cell growth. Cells were then washed with phosphate-buffered saline (PBS) 1X and air-dried overnight. Keratinocytes were then dyed with a 0.1% SRB (Sigma, Oakville, ON, Canada) solution, and linked SRB was solubilized with tris(hydroxymethyl)aminomethane (Tris) buffer. The absorbance was read at 540 nm using a microplate reader (SpectraMax Plus 384 Microplate Reader, Molecular Devices, San José, CA, USA). Experiments were performed on three biological replicates (cell population) and at least three technical replicates for each cell population (N = 3, n = 3). The antiproliferative potential and the concentration that inhibits 20% of cell growth (IC_20_) for each compound could then be determined, and the IC_20_ was used for further experiments on skin substitutes.

### 2.7. Viability Assay

The cell viability was evaluated using a 3-(4,5-dimethylthiazolyl-2)-2,5-diphenyltetrazolium bromide (MTT) assay. The MTT assay was used to measure cellular metabolic activity as an indicator of cell viability. This colorimetric assay is based on the enzymatic cleavage of a tetrazolium salt (MTT; yellow) to formazan crystals (purple) in metabolically active cells. Briefly, healthy and psoriatic keratinocytes at P2 were plated at 5 × 10^3^ cells/well in a 96-well plate on a feeder layer of irradiated 3T3 murine fibroblasts. On day 2, increasing concentrations of the compounds were added to the fresh media, and cells were incubated for 48 h (37 °C, 8% CO_2_). On day 4, wells were first washed with PBS 1X, and then a solution of 0.5 mg/mL MTT dye (Thiazolyl Blue Tetrazolium Bromide, Sigma, St. Louis, MO, USA) in sterile PBS 1X was added to the wells. The plate was incubated for 4 h (37 °C, 8% CO_2_), and the formazan was extracted with a fresh solution of isopropanol and hydrochloric acid (HCl). The absorbance was read at 570 nm using a microplate reader (SpectraMax Plus 384 Microplate Reader, Molecular Devices). Experiments were performed on three biological replicates (cell population) and at least three technical replicates for each cell population (N = 3, n = 3).

### 2.8. Skin Substitute Production

HSs and PSs were produced according to the self-assembly method, partially modified using 6-well plates [22,23]. Briefly, primary fibroblasts at P5 were seeded at 1.5 × 10^5^ cells/well and cultured for 26 days in DMEM supplemented with 50 μg/mL of (+)-sodium l-ascorbate (Sigma, St. Louis, MO, USA) until they formed manipulable sheets. Then, two fibroblast sheets were detached and superimposed to form the dermal equivalent. Dermal equivalents were incubated at 37 °C with 8% CO_2_ for two more days to allow the sheets to fuse and thus form the new layer. After this period, primary keratinocytes at P2 were seeded on the dermal equivalent at 1.2 × 10^6^ cells/dermal equivalent to form the epidermal layer and cultured for seven days in DMEMH supplemented with 50 μg/mL of (+)-sodium l-ascorbate (Sigma). Then, the skin substitutes were raised to the air-liquid interface and cultured with a medium lacking EGF to obtain a stratified epithelium representative of skin in vivo. On day 14 of culture at the air-liquid interface, HSs and PSs were treated or not with the compound stock solutions or with MTX diluted in culture medium three times per week for one week. After a total of 56 days of culture, skin substitute biopsies were taken and analyzed.

### 2.9. Histological Analyses

Skin substitute biopsies from each condition were fixed in HistoChoice solution and embedded in paraffin. Five-micrometer-thick sections were then cut and stained with Masson’s trichrome using Weigert’s hematoxylin, fuchsin-ponceau, and aniline blue dyes. The living epidermis thickness was measured with ImageJ software from histological photos. For the thickness measurements, three different cell populations (N = 3) were analyzed. For each of them, two representative skin substitutes per condition (n = 2) and three histological photos per substitute were analyzed, and 10 measurements per photo were performed for a total of 60 measurements per condition.

### 2.10. Immunofluorescence Staining

Skin substitute biopsies from each condition were embedded in Tissue-Tek Optimal Cutting Temperature (O.C.T.) Compound (Sakura Finetek, Torrance, CA, USA) and frozen in liquid nitrogen. Indirect immunofluorescence staining was performed on five-micrometer-thick cryosections fixed in acetone. The primary antibodies used were: mouse monoclonal anti-Ki67 (IgG) (dilution 1:400, Abcam, Cambridge, MA, USA), chicken polyclonal anti-keratin 14 (IgY) (dilution 1:500, BioLegend, San Diego, CA, USA), mouse monoclonal anti-involucrin (IgG) (dilution 1:1600, Sigma) and rabbit polyclonal anti-loricrin (IgG) (dilution 1:1000, Cedarlane, Burlington, ON, Canada). The saturation of non-specific sites was performed simultaneously with the labeling of the primary antibodies, using animal serum from the same species as the secondary antibodies used. The secondary antibodies used were: goat anti-mouse IgG (H + L) Alexa 488 (dilution 1:500, Life Technologies, Eugene, OR, USA), donkey anti-chicken IgG (H + L) Alexa 488 (dilution 1:500, Jackson ImmunoResearch, West Grove, PA, USA) and donkey anti-rabbit IgG (H + L) Alexa 488 (dilution 1:500, Life Technologies). Cell nuclei were labeled after the secondary antibody with the mounting medium DAPI Fluoromount-G (SouthernBiotech, Birmingham, AL, USA).

### 2.11. Statistical Analysis

Results were expressed as means ± standard deviation (SD). Living epidermis thickness measurements were analyzed with a one-way analysis of variance (one-way ANOVA) followed by a Tukey’s post hoc test (*p* < 0.05). Statistical analyses were performed with Prism software V5 (GraphPad Prism Software, San Diego, CA, USA).

## 3. Results

### 3.1. Antiproliferative Potential

A cell growth inhibition assay, precisely an SRB, was performed for each compound at increasing concentrations to determine their IC_20_. CPD1, CPD2 and CPD3 had an IC_20_ of 400, 150 and 350 μM respectively (Table 1). Moreover, the cellular metabolic activity was measured with an MTT assay as an indicator of cell viability in order to ensure that the cells were still viable despite the antiproliferative effect of the molecules. At the IC_20_, the cell viability for CPD1, CPD2 and CP3 was 97%, 94% and 97%, respectively (Table 1). Cultures treated with MTX, used as a control in this study, had a cell viability of 85% at a concentration of 734 μM. These concentrations were used in further experiments to treat HSs and PSs. Moreover, a DMSO control of 0.4% (*v*/*v*) was tested for these experiments and further ones, since the compounds were dissolved in 0.4% DMSO, but no difference was observed compared with their respective counterparts, thus confirming that a concentration of 0.4% DMSO was not toxic for skin cells and neither responsible for the effects observed (data not shown).

### 3.2. Skin Substitute Morphology

**Figure 2:**
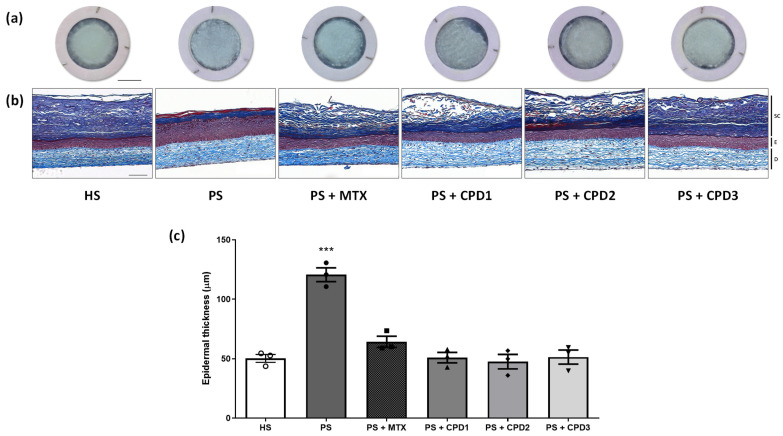
Effect of CPD1, CPD2 and CPD3 on skin morphology. (**a**) Macroscopic analyses and (**b**) histological cross-section with Masson’s trichrome staining of healthy substitutes (HSs); psoriatic substitutes (PSs); and psoriatic substitutes treated with methotrexate (MTX), quebecol (CPD1), ethyl 2,3,3-tris(3-hydroxy-4-methoxyphenyl)propenoate (CPD2) and bis(4-hydroxy-3-methoxyphenyl)methane (CPD3). Scale bars: (**a**) 1 cm; (**b**) 100 μm. SC: stratum corneum, E: epidermis, D: dermis. (**c**) Living epidermis thickness measurements from the histological analyses of HS (white circles), PS (black circles), PS + MTX (black squares), PS + CPD1 (black triangles), PS + CPD2 (black diamonds) and PS + CPD3 (black inverted triangles). Data are presented as means ± SD of the three biological replicates; N = 3, n = 2, 60 measurements per biological replicate. Statistical significance was determined using a one-way ANOVA followed by a Tukey’s post hoc test, *** *p* < 0.001.

Macroscopic analyses (Figure 2a) confirmed the integrity of the skin substitutes and showed the morphological difference between HSs and PSs. The epidermis appearance of HSs was smooth and uniform. The irregular and grainy appearance of PSs suggested hyperproliferation and an abnormal differentiation of keratinocytes.

Histological analyses of Masson’s trichrome-stained sections showed that PSs have a thicker epidermis compared with HSs (Figure 2b). Nevertheless, the different molecules restored the epidermal thickness of PSs to near normal. Indeed, the epidermal thickness of PSs treated with MTX, CPD1, CPD2 and CPD3 seemed to be more similar to the thickness of the HS living epidermis (Figure 2b). These observations could be confirmed with epidermal thickness measurements (Figure 2c). The PS living epidermis was significantly thicker than the HS living epidermis, with an average epidermal thickness of 121 ± 10 μm for PSs compared with 50 ± 6 μm for HSs (*p* < 0.001). When treated with the different molecules, PS epidermal thickness significantly decreased compared with PS without treatment (*p* < 0.001). Indeed, the epidermal thicknesses were 51 ± 7 μm, 48 ± 11 μm and 51 ± 10 μm when PSs were treated with CPD1, CPD2 and CPD3 respectively, which were not statistically different from HSs. Results for the different compounds are similar to what was observed with the comparative treatment MTX, suggesting that they are as effective as MTX on cell proliferation and differentiation, but at lower concentrations. When treated with MTX (PS + MTX), the epidermal thickness was 64 ± 8 μm, which is also statistically different from that of PSs (*p* < 0.001) and comparable to that of HSs. No significant difference was found between treated HSs and their respective counterparts indicating that the compounds do not affect healthy cells (data not shown).

### 3.3. Regulation of Hyperproliferation and Abnormal Differentiation

**Figure 3:**
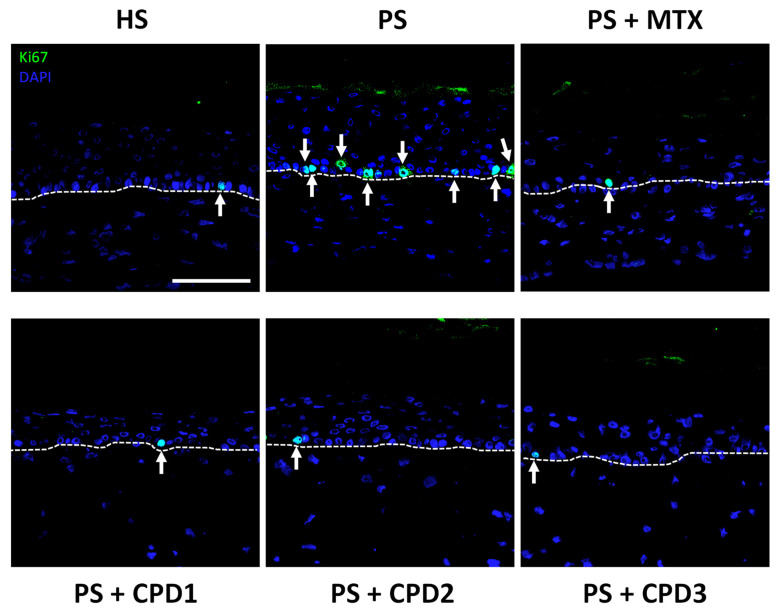
Immunofluorescence staining of a proliferation marker (Ki67, green). Expression of Ki67 (stained cells are indicated with arrows) in healthy substitutes (HSs); psoriatic substitutes (PSs); and psoriatic substitutes treated with methotrexate (MTX), quebecol (CPD1), ethyl 2,3,3-tris(3-hydroxy-4-methoxyphenyl)propenoate (CPD2) and bis(4-hydroxy-3-methoxyphenyl)methane (CPD3). Nuclei were stained with Hoechst (blue). The dotted white line represents the dermo-epidermal junction. Scale bar: 50 μm. Each staining is representative of three different cell populations.

Immunofluorescence staining was performed for proliferation (Ki67) and differentiation (keratin 14, involucrin and loricrin) markers in order to evaluate the antipsoriatic potential of the three compounds. For the proliferation marker Ki67 (Figure 3), results showed that more cells were stained for Ki67 in the basal layer of PSs compared with HSs, confirming the presence of hyperproliferation in PSs. The three compounds (CPD1, CPD2 and CPD3) restored the proliferative profile in PSs, since only a few basal cells were positive for Ki67 after treatments. The number of stained cells was closer to that of HSs, suggesting the regulation of the proliferative process after treatment with these compounds. These results are also similar to the comparative treatment MTX, which also decreased the number of stained cells in PSs and are in accordance with the decreased living epidermis thickness shown previously.

**Figure 4:**
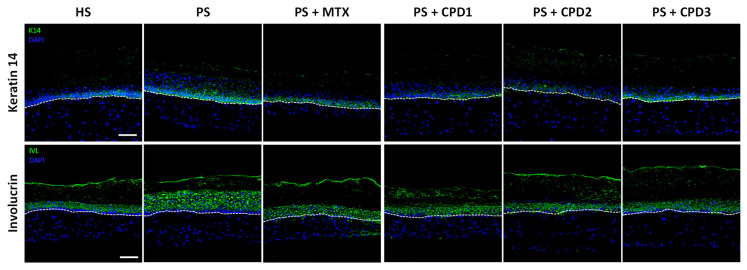
Immunofluorescence staining of early differentiation markers (green). Expression of keratin 14 (K14) and involucrin (IVL) in healthy substitutes (HSs); psoriatic substitutes (PSs); and psoriatic substitutes treated with methotrexate (MTX), quebecol (CPD1), ethyl 2,3,3-tris(3-hydroxy-4-methoxyphenyl)propenoate (CPD2) and bis(4-hydroxy-3-methoxyphenyl)methane (CPD3). Nuclei were stained with Hoechst (blue). The dotted white line represents the dermo-epidermal junction. Scale bars: 50 μm. Each staining is representative of three different cell populations.

Keratin 14 (K14), an early differentiation marker, is usually expressed in the basal layer. Thus, in pathological psoriatic skin, K14 expression is increased since the differentiation process is accelerated compared with healthy skin in vivo. In this study, PSs showed an overexpression of K14 and a more diffuse expression throughout the layers compared with HSs (Figure 4). When treated with either CPD1 or CPD2, K14 expression in PSs was restored to a similar HS phenotype compared with PSs without treatment, indicating the regulation of the abnormal differentiation process in psoriasis. As for CPD3, it did not seem to restore K14 expression in PSs as effectively as CPD1 and CPD2, suggesting a weaker effect on this differentiation marker. The MTX comparative treatment seemed to slightly decrease the expression of K14 in PSs compared with PSs without treatment, but not as effectively as CPD1 and CPD2, suggesting that those two compounds could be a better choice for the regulation of the differentiation process.

Another early differentiation marker, involucrin (IVL), was observed in order to assess the antipsoriatic potential of the compounds. IVL is expressed in the spinous layer in normal human skin, and its expression is usually increased in psoriasis. In PSs, IVL expression was indeed upregulated compared with HSs and expressed throughout the living epidermis (Figure 4). When treated with the studied compounds (CPD1, CPD2 or CPD3), PSs underwent a decrease in IVL expression to levels akin to a healthy profile. The expression was also restricted to the spinous layer as seen in HSs. This decrease is also comparable to the regulation of IVL expression observed in PSs treated with the comparative treatment, MTX.

**Figure 5:**
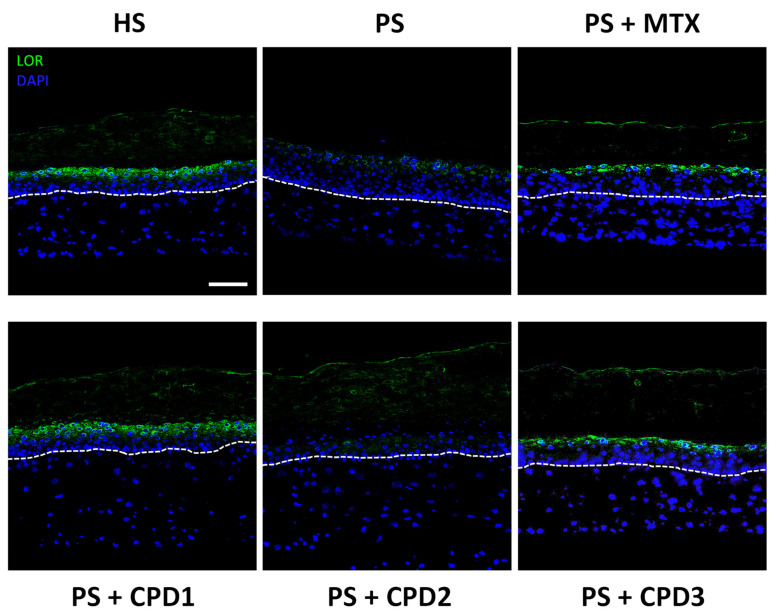
Immunofluorescence staining of a late differentiation marker (loricrin, green). Expression of loricrin (LOR) in healthy substitutes (HSs); psoriatic substitutes (PSs); and psoriatic substitutes treated with methotrexate (MTX), quebecol (CPD1), ethyl 2,3,3-tris(3-hydroxy-4-methoxyphenyl)propenoate (CPD2) and bis(4-hydroxy-3-methoxyphenyl)methane (CPD3). Nuclei were stained with Hoechst (blue). The dotted white line represents the dermo-epidermal junction. Scale bar: 50 μm. Each staining is representative of three different cell populations.

Finally, immunofluorescence staining was also used to observe loricrin (LOR), a late differentiation marker expressed in the granular layer. In psoriasis, late differentiation markers are usually underexpressed due to the accelerated differentiation process. As expected, LOR expression was lower in PSs than in HSs (Figure 5). Treatments with either CPD1 or CPD3 allowed the restoration of LOR expression in PSs to a level comparable to that of HSs. Unlike the two other compounds, CPD2 did not have any effect on the expression of this protein, suggesting that CPD2 does not play a role in the normalization of LOR expression. CPD1 and CPD3 restored LOR expression as effectively as MTX, the comparative treatment.

## 4. Discussion

Psoriasis is an inflammatory skin disease characterized mainly by an epidermal disorder including keratinocyte hyperproliferation and abnormal differentiation. Since psoriasis is an exclusively human disease and the etiology is still unknown, this disease is tedious to study and even more difficult to model. Over the years, the model of skin substitutes produced by the self-assembly method has demonstrated considerable accuracy with respect to the physiopathology of psoriasis, especially regarding the features mentioned above, namely the abnormal keratinocyte proliferation and differentiation processes. Proliferation markers, such as Ki67, keratin 5 and keratin 6, and early differentiation markers, such as involucrin, transglutaminase and keratin 14, are found to be overexpressed in this model, while late differentiation markers, such as filaggrin, loricrin and keratin 10, are underexpressed, which is comparable to the pathology in vivo [24,25,26,27]. This model developed by our group thus allows the evaluation of the antipsoriatic efficacy of novel compounds, which becomes an effective tool for the screening of lead compounds [18,25,28,29]. The healthy skin substitute model has even been used to evaluate the anti-aging efficacy of compounds, also confirming its response to different lead agents [30]. This model has thus proved its usefulness in the search for new antipsoriatic compounds, considering that psoriasis is a complex disease without a curative treatment. Niehues and van den Bogaard even consider that the self-assembly method with psoriatic cells (keratinocytes and fibroblasts) isolated from patients’ lesions is one of the most representative models to date, as it displays a large number of psoriatic features [31]. 

Maple sap and maple syrup have recently attracted serious interest in the research community, mainly due to their high phenolic content. It is only recently that quebecol, a phenolic compound formed during the heating process required to transform the sap into syrup, was identified in maple syrup [11]. In order to evaluate the biological activity of quebecol, it was investigated for its potential as a chemotherapeutic agent, showing an antiproliferative effect on cancer cell lines [13]. Quebecol and some of its derivatives were also reported to have in vitro anti-inflammatory activity as shown by their inhibition of the secretion of proinflammatory cytokines (interleukin-6 (IL-6) and tumor necrosis factor alpha (TNF-α)) and the reduction in the activation of the NF-κB transcription factor in LPS-stimulated human macrophages, which are involved in the pathology of psoriasis [14]. These previous results led our group to believe that these molecules could have antipsoriatic potential and inspired the study of quebecol and its derivatives in our skin model. IL-6, a major mediator in the inflammatory response, is produced by both keratinocytes and fibroblasts in psoriatic skin [32]. Moreover, IL-6-treated normal keratinocytes showed increased proliferation, suggesting the involvement of this proinflammatory cytokine in the hyperplasia of psoriasis [33]. Another contributor to the formation of psoriatic skin lesions is TNF-α. This proinflammatory cytokine primarily secreted by keratinocytes and fibroblasts upon stimulation is present at increased levels in psoriatic skin and is suspected to be involved in the development of papillomatosis and acanthosis in psoriatic lesions [34,35,36,37]. Thus, the results obtained in our study with quebecol and its derivatives indicate that the acanthosis and hyperplasia features of psoriasis could be mediated via the inhibition of IL-6 and TNF-α production. NF-κB, a transcription factor, is a key regulator in the pathogenesis of psoriasis [38]. Indeed, the activity of NF-κB is known to be increased in psoriasis, and it is believed to be involved in the crosstalk between immune cells and keratinocytes and to regulate the activity of numerous proinflammatory mediators [38,39,40,41,42]. NF-κB would also be involved in several other biological processes, such as cellular proliferation, differentiation and apoptosis, and could therefore be a key mediator in the hyperproliferative and abnormal differentiation processes of psoriasis [38,43]. Moorchung et al. found that the expression of NF-κB in the epidermis and basal cells would be responsible for at least two pathological features of psoriasis, namely hyperplasia and the infiltration of immune cells [41]. According to these studies, quebecol and its derivatives could mediate the activation of NF-κB since the compounds reduced the severity of certain features of the psoriatic phenotype of our skin substitutes, especially hyperplasia.

Despite the biological activities of these compounds strongly suggesting an antipsoriatic potential, in-depth studies of their efficacy on skin substitutes representative of pathological skin in vivo had never been conducted before. An antipsoriatic screening of these compounds was performed in this study, and the three compounds, CPD1, CPD2 and CPD3, presented good antipsoriatic potential with a regulation of the proliferation and differentiation processes. Results showed that all three compounds have a relatively good IC_20_ compared with MTX (at its therapeutic dose); CPD2 has the lowest concentration (150 μM) and CPD1 has the highest (400 μM). At their IC_20_, all compounds preserved cell viability, which is important for an antipsoriatic treatment. Indeed, the antiproliferative strategy is one approach for the development of treatments for psoriasis [44]. Treatments need to regulate the growth of psoriatic cells without altering their integrity, which was the result obtained in this study with the three compounds. The compounds were even comparable to the positive control MTX for certain differentiation markers, and CPD1 seemed even better than MTX. These results and those on the epidermal thickness are in accordance with previous studies on quebecol’s antiproliferative effect on cancer cell lines [13]. Indeed, the three compounds effectively reduced the living epidermis thickness of the treated PSs, which had a living epidermis significantly thicker than that of HSs. This antiproliferative activity was further confirmed by Ki67 staining. In PSs, the number of stained cells in the basal layer was significantly higher compared with HSs, which confirmed the hyperproliferative characteristic of the model. PSs treated with CPD1, CPD2 and CPD3 had a similar number of stained cells as HSs and PSs + MTX, showing their considerable potential for regulating the hyperproliferation of psoriatic keratinocytes. 

In addition to their antiproliferative properties, the three compounds displayed a significant ability to regulate the differentiation process in psoriasis. Indeed, the differentiation of the psoriatic epidermis is highly dysregulated, which is reflected by a dysregulation of key differentiation markers. Early differentiation markers tend to be overexpressed in native psoriatic skin, while late differentiation markers are underexpressed. Immunofluorescence staining of the early differentiation markers K14 and IVL highlighted the ability of quebecol and its derivatives to regulate epidermal differentiation as shown by a decrease in these markers’ expression in treated psoriatic substitutes. During the epidermal differentiation process, different types of keratins are synthesized. K14, along with K5, is expressed by basal keratinocytes and is found to be upregulated in lesional psoriatic skin compared with normal skin [45]. As for IVL, it is a major component of the cornified cell envelope synthesized by squamous epithelial cells, particularly in the stratum spinosum [46,47]. With the accelerated differentiation process in psoriasis, the expression of precursor proteins such as IVL is increased and persistent throughout the suprabasal layers [48]. The three compounds had very similar effects on these markers, which were also akin to the effect of MTX, except that CPD3 had a weaker effect on the regulation of K14. Expression of the late differentiation marker LOR was reestablished by CPD1 and CPD3, while CPD2 did not seem to modulate this marker’s expression. LOR is produced by granular cells in the stratum granulosum, and with maturation, the cells release their contents into the cornified layer to be incorporated into the cornified cell envelope [49]. Since there is an accelerated differentiation process in psoriasis, this pathology is characterized by hypogranulosis (and even agranulosis), i.e., the absence of the stratum granulosum, resulting in the downregulation or absence of late differentiation markers such as LOR [50]. The modulation of differentiation marker expression by the three compounds shows their potential in the regulation of the differentiation process, leading notably to the re-establishment of all the epidermal layers, including the granular layer. Taken together, these results suggest that all three compounds can regulate the differentiation of the psoriatic epidermis, with CPD1 having the effects that most closely resemble MTX.

**Figure 6:**
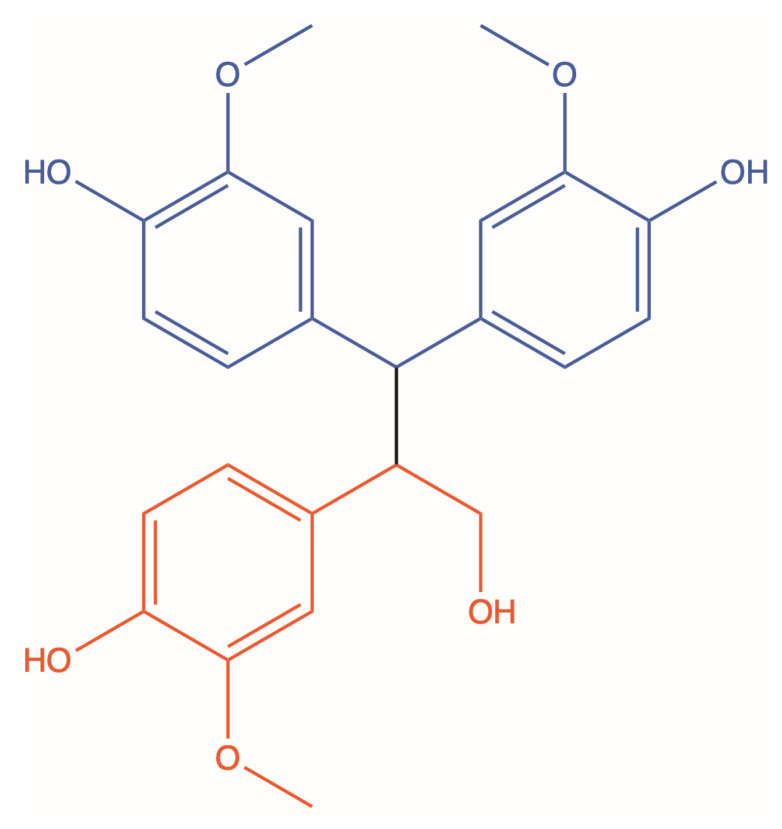
North and South substructures of quebecol. The North substructure is colored in blue, and the South substructure is colored in red.

In this study, quebecol and two of its derivatives were investigated for their antipsoriatic activities. Quebecol’s structure can be divided into two parts: the “North” substructure and the “South” substructure (Figure 6). It was previously shown in a structure–activity relationship study that the North substructure of quebecol, corresponding to CPD3 in our study, is responsible for its anti-inflammatory activity [14]. Indeed, this substructure was found to be the most efficient inhibitor of the proinflammatory cytokines studied, and its cytotoxicity and activity towards LPS-induced NF-κB activation were similar to those observed for quebecol. Conversely, the South substructure of quebecol displayed no significant anti-inflammatory properties, suggesting that this region is not a major contributor to the anti-inflammatory activity of quebecol. Interestingly, a parallel can be drawn between this previously reported structure–activity relationship and our results. Indeed, CPD1 (quebecol) and CPD3 had a very similar IC_20_ and cytotoxicity, as well as similar effects on the proliferation and differentiation processes of the psoriatic keratinocytes in our 3D skin model. In this sense, it is possible that the antipsoriatic activity of quebecol originates from its North substructure. Moreover, the South substructure showed no significant antiproliferative potential in the SRB assay, which is why it was not further investigated (data not shown). However, CPD3 had a weaker effect on K14 expression than CPD1, suggesting that the other substructure of quebecol, or at least another functional group, may be important for the antipsoriatic effect. It was also reported that the triarylethene analog of quebecol, corresponding to CPD2 in this study, had the most pronounced effect of all studied molecules on LPS-induced NF-κB activation [14]. As mentioned previously, NF-κB is known to regulate the epidermal hyperplasia in psoriasis, suggesting that the results obtained with CPD2 on the regulation of the hyperproliferation and abnormal differentiation could be mediated via NF-κB [38]. Taken together, our results correlate well with the previously reported structure–activity relationship, showing that the “North” substructure might be responsible for the antipsoriatic activity of quebecol.

## 5. Conclusions

The improvement in the psoriatic phenotype of PSs treated only three times with the compounds throughout a week confirmed the interesting antipsoriatic potential of these compounds, as they seemed to regulate the proliferation and differentiation processes according to Ki67, keratin 14, involucrin and loricrin immunofluorescence staining. However, CPD3 failed to restore K14 expression in treated PSs, while CPD2 failed to restore LOR expression, indicating that only CPD1 had full efficacy on the abnormal differentiation process. In light of these results, CPD1, namely quebecol, seemed to have the most promising antipsoriatic efficacy. Further studies such as anti-inflammatory activity on psoriatic skin substitutes could be beneficial in order to assess its practical potential as a psoriasis treatment. Clinical studies would also be needed to confirm its efficacy as an active ingredient in dermatological products.

## Figures and Tables

**Table 1:** Cell growth inhibition concentration (IC_20_) and cell viability at the IC_20_ for each tested compound.

Compound	IC_20_ (μM)	Cell Viability (%)
CPD1	400	97
CPD2	150	94
CPD3	350	97
MTX	734 ^1^	85

^1^ MTX concentration is not the IC_20_, but rather the calculated concentration generally used as a treatment.

## Data Availability

The data presented in this study are available in the article.
